# Identification of a gene-expression predictor for diagnosis and personalized stratification of lupus patients

**DOI:** 10.1371/journal.pone.0198325

**Published:** 2018-07-05

**Authors:** Yan Ding, Hongai Li, Xiaojie He, Wang Liao, Zhuwen Yi, Jia Yi, Zhibin Chen, Daniel J. Moore, Yajun Yi, Wei Xiang

**Affiliations:** 1 Department of Dermatology, Hainan Provincial Dermatology Disease Hospital, Haikou, China; 2 Pediatrics, The Hainan Affiliated Hospital of University of South China, Haikou, China; 3 Department of Nephropathy, Children’s Medical Center, The Second Xiangya Hospital, Central South University, Changsha, China; 4 Department of Cardiology, Hainan General Hospital, Haikou, China; 5 Department of Medicine, University of North Carolina at Chapel Hill, Chapel Hill, North Carolina, NC, United States of America; 6 Department of Microbiology and Immunology, University of Miami Miller School of Medicine, Miami, FL, United States of America; 7 Departments of Pediatrics and Pathology, Microbiology, and Immunology, Vanderbilt University, Nashville, TN, United States of America; 8 Department of Medicine, Vanderbilt University Medical Center, Nashville, TN, United States of America; 9 Department of Pediatrics, Maternal and Child Health Care Hospital of Hainan Province, Haikou, China; IMAGINE, FRANCE

## Abstract

Systemic lupus erythematosus (SLE) is an autoimmune disease characterized by a wide spectrum of clinical manifestations and degrees of severity. Few genomic biomarkers for SLE have been validated and employed to inform clinical classifications and decisions. To discover and assess the gene-expression based SLE predictors in published studies, we performed a meta-analysis using our established signature database and a data similarity-driven strategy. From 13 training data sets on SLE gene-expression studies, we identified a SLE meta-signature (SLEmetaSig100) containing 100 concordant genes that are involved in DNA sensors and the IFN signaling pathway. We rigorously examined SLEmetaSig100 with both retrospective and prospective validation in two independent data sets. Using unsupervised clustering, we retrospectively elucidated that SLEmetaSig100 could classify clinical samples into two groups that correlated with SLE disease status and disease activities. More importantly, SLEmetaSig100 enabled personalized stratification demonstrating its ability to prospectively predict SLE disease at the individual patient level. To evaluate the performance of SLEmetaSig100 in predicting SLE, we predicted 1,171 testing samples to be either non-SLE or SLE with positive predictive value (97–99%), specificity (85%-84%), and sensitivity (60–84%). Our study suggests that SLEmetaSig100 has enhanced predictive value to facilitate current SLE clinical classification and provides personalized disease activity monitoring.

## Introduction

Systemic lupus erythematosus (SLE) is a chronic systemic autoimmune disease that predominantly affects young women. It is characterized by heterogeneous clinical manifestations with varying degrees of severity punctuated by alternating phases of remission and flares [1]. Patients classically produce high autoantibody titers that form immune complexes that are deposited in many different organs such as the skin, joints, and kidneys causing cumulative damage over time [2].

Given its wide variety of relapsing-remitting symptoms, lupus is typically difficult to diagnose. To complicate matters, there is no laboratory test that can definitively identify the illness. As a result, it can take years for a diagnosis to be made. Disease activity (DA) is also difficult to measure. One method is using any of six validated composite scores, one of which is the SLE Disease Activity Index (SLEDAI) [3]. Because SLE is heterogeneous, not all manifestations are included in the SLEDAI, making reliable patient assessment challenging. Therefore, there is a critical need for a biomarker to detect, monitor, and stratify individual patients with SLE.

The use of gene expression microarrays in clinical research has led to the establishment of biomarker signatures. SLE patients display unique blood transcriptional signatures linked to type I interferon (IFN) and granulocytes [4–7]. Preliminary work suggests that these signatures correlate with SLE disease status and DA [6]. Most studies have focused on IFN-induced transcripts or proteins as biomarkers [7–9].

The value of this approach to discover stable disease markers has been questioned [10]. One common weakness is that the traditional approach employs single study-based signatures that are derived from small sample-size and lack cross-validation making data interpretation and application difficult. This limitation underscores the need to assess larger cohorts, to use unbiased approaches that incorporate all elements of the signature, and to account for disease heterogeneity during data interpretation.

These limitations can be overcome by combining related but independent studies into a meta-analysis forming a larger sample size with lower false discovery rates. We have developed and utilized a robust meta-analysis approach called EXALT(EXpression AnaLysis Tool) for gene expression profile studies from thousands of Gene Expression Omnibus (GEO) and published breast cancer datasets [11–13]. A gene expression signature as defined by EXALT is a set of significant genes with their corresponding statistical scores and gene expression direction codes (up or down). We have previously used this approach to discover a novel and conserved gene expression signature predictive of metastasis risk in multiple cancers [13].

The present work describes the implementation of our unique EXALT approach for the meta-analysis of blood microarray transcriptional profiles on SLE. To this end, we aimed to identify a meta-signature (SLEmetaSig100) that correlates SLE status and DA from thirteen training data sets. We then validated the SLEmetaSig100 in two independent test data sets to determine its correlation with SLE and DA and prospective predictive value of SLE disease at the individual patient level. This enabled patient stratification based on a personalized transcriptional immunomonitoring signature correlating with DA in each patient.

## Materials and method

### Publicly available data sets and signatures

Subjects were recruited by the individual studies. Clinical diagnosis and demographic characteristics of anonymized SLE patients were confirmed and summarized in the previously published studies (S1 Table). EXALT is a database that holds original study descriptions, sample phenotypes, curated gene expression datasets, as well as thousands of gene expression signatures extracted from the GEO and other published studies. These meta-data and gene-expression profiles are encoded in a searchable format to form the basis of our data analysis[13]. With EXALT database, we are able to search data sets based on similar sample phenotypes and study design, subsequently identifying fifteen gene-expression microarray data sets on various SLE phenotypes. These were then further divided into thirteen training sets and two testing data sets. Two test data sets were selected for their large sample size and comprehensive clinical information.

Whole blood PBMC or T cell subset samples from the training data sets (n = 1,869) were grouped by their clinical attributes and study designs. Based on the existing sample group description in the published studies, each data set had at least two groups of samples including normal healthy controls and various SLE related phenotypes, and/or molecular markers such as a lupus flare, low or high disease grade activities, TLR and IFN gene expression levels (S1 Table). Two or more groups per dataset were needed to generate statistical comparisons. A total of 167 SLE gene signatures from all possible pairwise group comparisons were generated accordingly [11].

One of the signatures (PMID: 16777955) in the training set (S1 Table) was derived from a mouse model (Low vs Overexpression of TLR7). In order to define corresponding human TLR7 signature for cross-species meta-analysis, we used NCBI Gene and NCBI HomoloGene databases to translate mouse array probesets to human homolog gene symbols as we described before[14, 15].

### Identification of meta-signature

We used EXALT in an iterative manner (iterative EXALT) [13] to conduct a data similarity-driven clustering analysis of the 167 SLE gene-expression signatures and to elucidate a common transcriptional signature in SLE studies. This iterative EXALT process started with all-versus-all signature similarity searches, resulting in signature clusters. More specifically, each of the 167 SLE signatures was searched against other 166 signatures to bring homologous signatures together by their intrinsic similarities. This process ‘‘grouped” or ‘‘clustered” the thirteen signatures together based on their similarities (i.e. gene names, expression directions, and confidence levels) to form the SLE signature cluster. We focused on this cluster because their phenotypes were clearly related to known SLE disease status or pathogenesis such as SLE flare activity, IFN production, or TLR7 expression.

In the cluster, each of the thirteen signatures comprised several hundred genes with various overlapping signature genes. In order to identify a recurrent and concordant gene expression pattern in the SLE signature cluster, all signature genes were assembled together to form a synthetic signature (SLEmetaSig). The top 100 genes (SLEmetaSig100), as determined by ranking their frequency of recurrence and gene expression profile concordance, were identified using the method previously described [13].

### Prospective prediction of SLE status

We constructed a centroid-based reference signature associated with known SLE status and the SLEmetaSig100 signature values from the thirteen training datasets using the method described before [16].

Two GEO datasets (GSE65391 and GSE11909) were used as validation data sets to test the predictive ability of SLEmetaSig100. The 1,171 testing SLEmetaSig100 signatures were made from 92 healthy subjects and 1,079 SLE samples (211 patients) [6, 17].

By performing a Spearman’s rank correlation between the reference signature and individual gene-expression profiles in test datasets, we were able to determine SLE status of individual patients from the two test data sets.

For each testing sample, a Spearman rank correlation value between the reference SLEmetaSig100 profile and the test sample profile was calculated. The sample is considered to be ‘SLE’ if the correlation value with the reference was equal to or above the predefined threshold value (0.3)[18]. The sample was considered healthy otherwise.

### Statistics

An EXALT built-in statistical approach was used to assess signature similarity of training data sets. To evaluate of SLEmetaSig100 retrospective classification, we used *Mann-Whitney U test* to examine the difference in mean numbers SLE subjects between two SLEmetaSig100 classified groups. We used Fisher’s Exact Test to compare prospective prediction rates of SLEmetaSig100 in two test data sets.

Hierarchical clustering and Spearman's rank correlation were performed and visualized using the TIGR MeV [19]. Unsupervised hierarchical clustering based on average linkage was conducted to group the patient samples. The group assignments were based on the first bifurcation of the clustering sample dendrogram [20]. The Spearman rank correlation was used to measure the correlations between the reference signature and individual testing profiles in the two test data sets.

Prospective SLE prediction in test data sets was compared with actual clinical diagnosis. The primary predictive endpoint was SLE diagnosis or SLE disease activity (DA) for the validation cohort. The predictive performance was assessed using the derived positive predictive value (PPV), the negative predictive value (NPV), sensitivity, and specificity.

Receiver operating characteristic (ROC) analysis was performed to determine the sensitivity and specificity of SLEmetaSig100 predictions and the area under the ROC curve. ROC analyses were performed using R, version 3.3.3.

## Results

### Identification of human SLE meta-signature

In the past two decades, a large number of gene-expression studies have been reported and deposited in public domain including GEO, PubMed, and EXALT signature database [11]. EXALT manages signatures that are derived from all possible comparisons of each data sets including all possible experimental and disease conditions.

To avoid the weaknesses of single study-derived signatures and to better utilize the available gene expression data from independent studies, we have developed a meta-analysis strategy called EXALT. EXALT is essentially a database containing thousands of gene expression signatures extracted from published studies that enables signature comparisons [13]. We have extracted signatures from over 1,500 data sets representing over 22,367 samples from various diseases and experimental conditions collected from NCBI GEO and PubMed [11]. Searching through signature similarity sample phenotypes, and design information, we identified fifteen data sets on SLE gene expression profiling and then partitioned them into thirteen training and two testing data sets with 1,869 and 1,171 samples, respectively (S1 Table). From the thirteen training data sets, we extracted 167 gene-expression signatures.

Some of these 167 gene signatures are biologically related to SLE disease status. There are inherent limitations for any individual profiling study such as small sample size relative to the large number of potential gene probes, limitations of technological platforms, sample variation, and bioinformatics or statistical method bias. To overcome these problems, we implemented a meta-analysis approach (iterative EXALT) that combines individual transcriptional profiling signatures to deduce a common transcriptional signature across studies (SLEmetaSig100).

**Fig 1:**
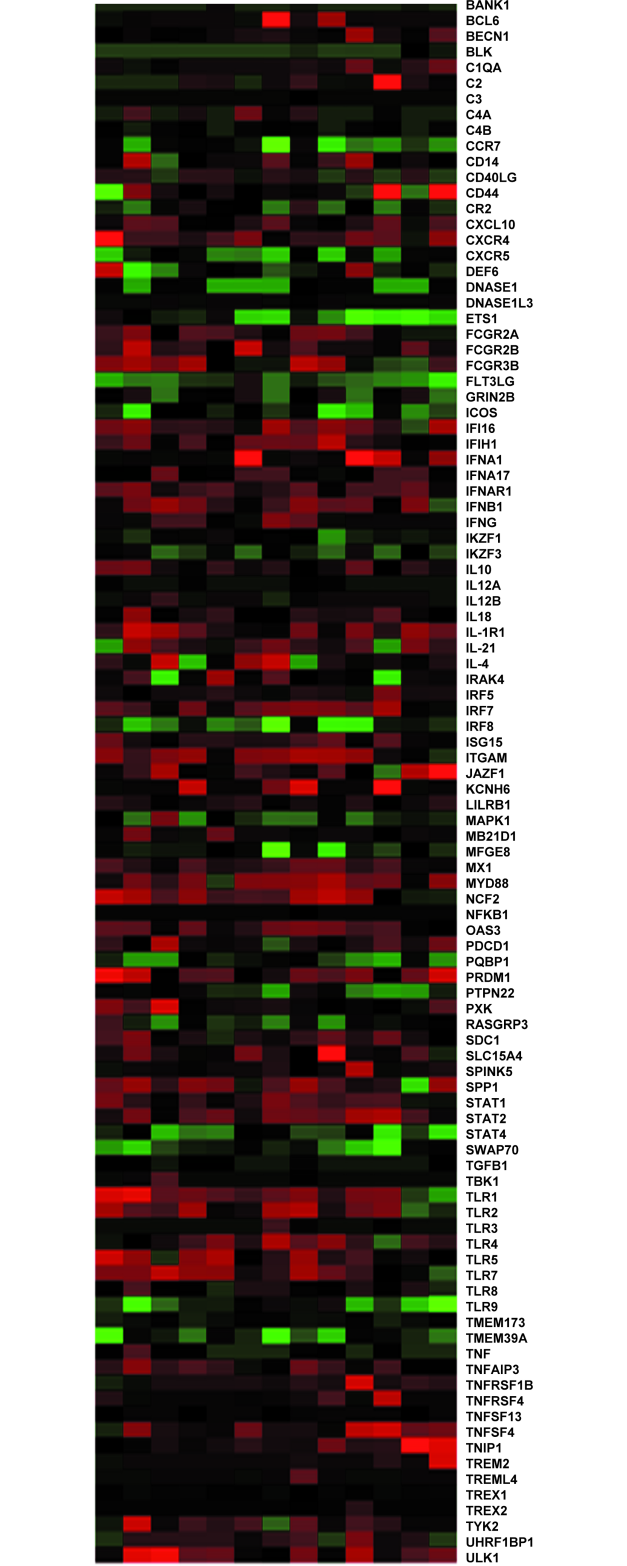
Co-expression analysis of the 100 meta-signature genes from the SLE training data sets. Using EXALT meta-analysis, thirteen SLE signatures in columns with similar phenotypes indicated in S1 Table were displayed in a heat map with 100 genes (SLEmetaSig100) displayed in rows. The colors in the meta-heat map represent the direction of differential gene expression within a given transcriptional profile (red for up, green for down, and black for a missing match). Color intensity reflects the confidence levels of differential expression in the signatures.

This conserved profile (SLEmetaSig100) was derived from 1,869 patient samples from thirteen individual SLE studies (Fig 1, S1 Table). The expression directions (up or down-regulation) and the function of the genes enriched in SLEmetaSig100 are displayed in Fig 1 and summarized in S2 Table. These genes are mechanistically involved in the pathogenies of SLE or other autoimmune diseases. Thus, SLEmetaSig100 likely represents genes involved in SLE disease pathogenesis.

### Pathways analysis of SLE signature genes

To determine pathways that SLEmetaSig100 may be involved in, we used KEGG Pathway database (http://www.genome.jp/kegg) and its analysis tool[21].

The pathway analysis results suggested that SLEmetaSig100's genes involved in numerous pathways such as Toll-like receptor signaling pathway, NF-kappa B signaling pathway, and cytokine-cytokine receptor interaction network.

**Fig 2:**
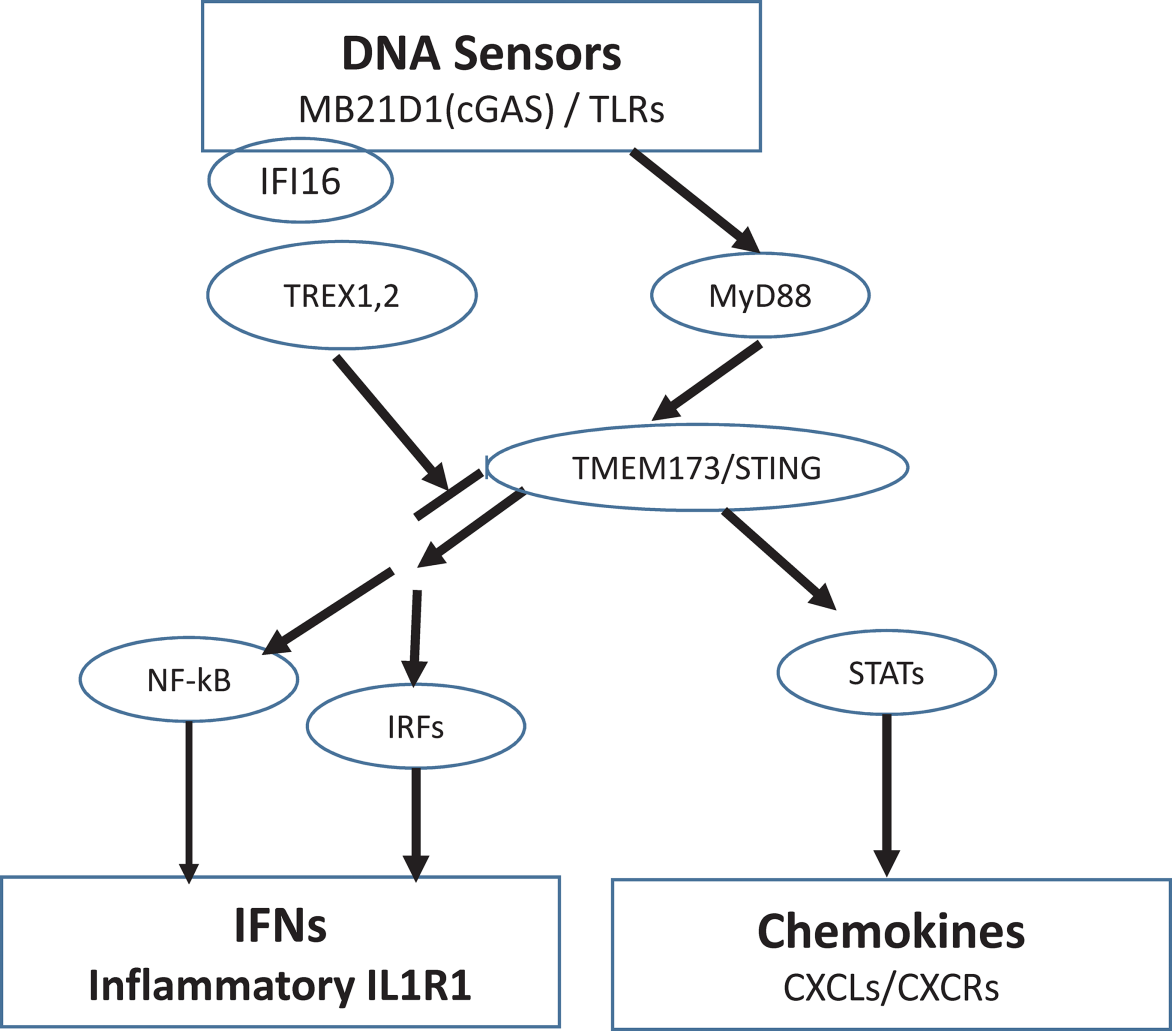
Pathway analysis of SLEmetaSig100 genes. Pathway analyses of SLEmetaSig100 genes identified genes involving DNA sensors and the cytokines constructing an innate immune DNA-sensor model. SLEmetaSig100 genes are marked in white circles or rectangles. DNA sensors include MB21D1(cGAS), multiple TLR genes, TMEM173/STING, and IF16 genes. In the Toll-like receptor signaling pathway, the stimulation of DNA sensor genes by microbe-derived and/or host DNA are positively regulated by MYD88 and TMEM173/STING genes and negatively regulated by TREX1 and TREX2 genes. The downstream cytokine-cytokine receptor interaction genes include NF-kappa B signaling pathway mediated IFNs, inflammatory cytokines (e.g. IL1R1), and STATs mediated chemokines (CXCL and CXCR genes).

We categorized these pathway genes into two major functional categories (DNA sensors and cytokine genes) and constructed an innate immune DNA-sensor model of SLEmetaSig100. A cartoon depicting genes and their relationship in this model is shown in Fig 2.

DNA sensor genes such as cyclic GMP-AMP Synthase (MB21D1 or cGAS), IFI16, and Toll-like receptors (TLRs) are upstream stimulators in Toll-like receptor signaling pathway (S1 Fig), NF-kappa B signaling pathway (S2 Fig), and cytokine-cytokine receptor interaction network (S3 Fig). These pathways work synergistically to mount an immune response to either a pathogen or cellular or tissue damage. The role of TLRs in the onset of autoimmune pathologies has been effectively addressed in murine models of SLE [22]. DNA sensors have been demonstrated to be essential for inducing inflammatory genes, *e*.*g*. IFN-β expression [23]. IFN gene over-expression patterns have been reported in SLE patients [24]. SLE can be distinguished by a remarkably homogeneous gene expression pattern with overexpression of granulopoiesis-related and interferon (IFN)-induced genes [5]. Thus, it is possible that changes in expression of DNA sensors, TLRs, NF-kappa B genes, and cytokines are indicators for SLE pathogenesis.

To further study the characteristics of SLEmetaSig100, we compared SLEmetaSig100 with some other known SLE signatures (Table 1). Unlike many other SLE signatures[5, 8, 9, 17], SLEmetaSig100 was identified based on much larger training sets from a meta-analysis of thirteen training data sets and two independent test data sets for validation.

**Table 1:** SLE signature comparison.

Signature	SLEmetaSig100	Plasmablast	IFN	IFIGs	IFNr
**Gene Number**	100	9	10	3	3
**Overlapping Genes**	100	0	1	0	1
**Training Set Size**	1869	649	39	NA	NA
**Test Set Size**	1171	12	0	127	93
**Method**	meta-analysis	modular	Hochberg	scores	scores
**SLE Association**	DA	DA	DA	renal	No
**Retrospective Stratification**	Yes	Yes	Yes	Yes	No
**Prospective Prediction**	Yes	ND	ND	ND	ND

NOTE: ND, not done.

When signature genes were analyzed by an overlapping analysis, we found that there were very few common genes between SLEmetaSig100 and others. For example, only one common gene (1%) was found between SLEmetaSig100 and the other two signatures (IFN signature[5] and IFNr signature[9]). There is no common gene between SLEmetaSig100 and other known signatures such as plasmablast signature[17] and IFN-induced genes (IFIGs) signature[8]. Because of discrepant patient populations and signature extraction methods (Table 1), the result suggests that the five previously reported SLE studies identified different blood transcriptional signatures. Despite this small amount of overlap in gene composition, the major functional component of these signature genes are all linked to interferon (IFN) and/or IFN-induced genes (IFIGs) (Table 1).

### Stratification of SLE patient by the SLE 100-gene signature

**Fig 3:**
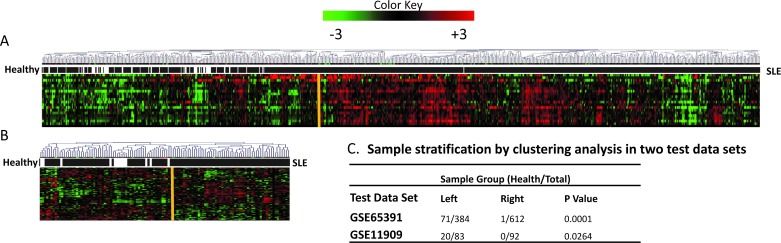
Stratification of human samples by clustering the SLEmetaSig100 meta-profiles. Meta-heat maps from unsupervised hierarchical clustering depict meta-profiles in two test data sets, (A) GSE65391 and (B) GSE11909. In each panel, the gene expression patterns from one given test data set are represented in rows and samples are clustered in columns. The colors in the heat map represent the direction of differential gene expression within a given transcriptional profile (red for up, green for down, and black for a missing match). Color intensity reflects the confidence levels of differential expression. Sample groups in columns are determined by the top hierarchy nodes of dendrograms (yellow bar) into left and right sample groups. The sample phenotype patterns underneath each sample dendrogram panel are indicated by black (SLE) and white (healthy) bars. The classification of healthy samples from total samples (healthy/total) by SLEmetaSig100 profiles between two sample groups (left and right) and statistic tests (Mann-Whitney U test) results are listed in a table (C).

In order to validate the correlation between SLEmetaSig100 and SLE disease, we surveyed gene expression profiles of SLEmetaSig100 from two independently published transcriptional profiling studies [GSE65391and GSE11909] performed on normal versus SLE disease states (Fig 3).

Both genes and samples were clustered by their gene-expression profiles. The sample classifications were determined based on the SLEmetaSig100 genes expression patterns (yellow bars in Fig 3A and 3B). Meta-heat maps were used to illustrate SLEmetaSig100 gene expression profiles and its capability to classify 996 (Fig 3A) and 175 human samples (Fig 3B).

Based on the meta-heat maps, we were able to classify human samples into left and right sample groups (yellow bar) in the dendrograms based on differential gene expression patterns. The associated sample phenotypes, either healthy (white bar) or SLE (black bars), were also grouped in each dataset. We observed that healthy samples were enriched in the left group of both testing data sets (Fig 3A and 3B). The results demonstrated that SLEmetaSig100 could stratify human samples into two groups: the normal healthy enriched sample groups (left) and the SLE dominant sample group (right).

More specifically, in the SLE dominant sample groups, there was only one (out of 72 total) normal sample in GSE65391(Fig 3A) and none (out of 20 total) in GSE11909 (Fig 3B), while remaining normal samples (71 in GSE6539 and 20 in GSE11909) were grouped in the normal sample groups. Thus, there were significantly fewer normal samples in SLE dominant sample groups than those in the normal sample groups of two test data sets (P = 0.0001 and 0.0264, Fig 3C). These results suggest that with known clinical SLE information SLEmetaSig100 is capable of stratifying testing samples into a normal health group and a SLE group.

### Personalized SLE prediction in individual patients using SLEmetaSig100

SLE is a heterogeneous disease that cannot be diagnosed by a single symptom or lab test. Personalized prediction of SLE status by comparing a test sample profile to a reference SLEmetaSig100 signature may provide a new method to facilitate clinical diagnosis.

We leveraged SLEmetaSig100 to determine whether it can distinguish individual SLE patients from normal healthy subjects and to uncover associated disease activity (SLEDAI) when clinical SLE information is masked in test data sets.

**Fig 4:**
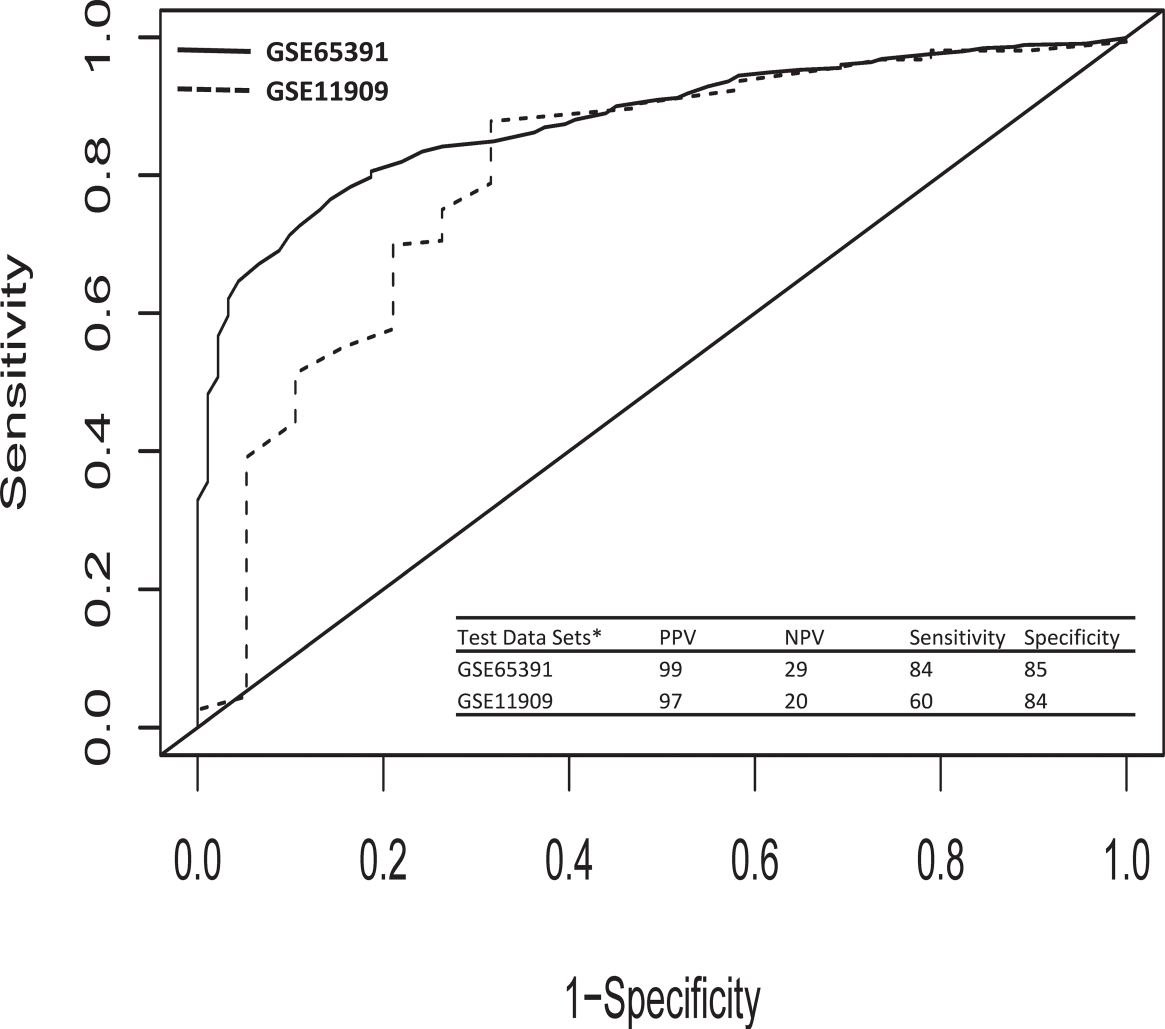
Receiver operating characteristic (ROC) curves for SLEmetaSig100. Area under receiver operating characteristic curve (AUC) for performance of SLEmetaSig100 were calculated in two testing cohorts, GSE65391(solid line) and GSE11909(dash line), and SLEmetaSig100 significantly outperforms the random prediction of SLE disease (AUC, 0.89 in GSE65391 and 0.85 in GSE11909). The sub-table shows SLEmetaSig100 prediction performance in two test datasets. *Note: SLE prediction by SLEmetaSig100 in two test data sets was examined by Fisher Exact test (P value = 1.48E-36).

To evaluate the ability of SLEmetaSig100 to predict SLE, we divided 1,171 testing samples to be either predicted healthy or predicted SLE. Those results were then compared to their actual clinic diagnoses. The derived positive predictive value (PPV), the negative predictive value (NPV), sensitivity, and specificity were then calculated and compared accordingly. While assessing the SLEmetaSig100 prospective prediction results with the actual clinic diagnoses, we found that SLEmetaSig100 could significantly correctly predict SLE in two independent cohorts (sub-Table in Fig 4, P = 1.48E-36). The prospective predictions using the SLEmetaSig100 centroid model showed comparable results to those obtained using the unsupervised clustering-based retrospective prediction at group level (Fig 3). However, the centroid model can further prospectively apply to individual patients with high PPV (97%-99%), specificity (85%-84%), and sensitivity (60–84%) (sub-Table in Fig 4).

SLEmetaSig100’s ability to discriminate between normal healthy subjects and SLE patients was also evaluated by ROC analysis in two test data sets (GSE65391 and GSE11909). The ROC results are comparable displaying areas under the curve (AUC) of 0.89 (GSE65391) and 0.85 (GSE11909), respectively, indicating that SLEmetaSig100 has reliable performance in heterogeneous patient populations. When SLEmetaSig100 prediction was implemented with centroid model, the GSE65391 test resulted in a specificity of 85% and a sensitivity of 84%. The GSE11909 test displayed a specificity of 84% and a sensitivity of 60% (sub-Table in Fig 4).

To determine the association between SLE DA and SLEmetaSig100 prediction, we used the SLE samples from the training set with a known SLE disease activity index (SLEDAI). These samples were classified into three DA group scores: S1 (SLEDAI: 0–2), S2 (SLEDAI:3–7), or S3 (SLEDAI > 7). From this, we constructed three DA reference signatures (DA1, DA2, and DA3). Using the same SLE prediction process, we computed three correlation scores between each test sample and three reference DA signatures. The predicted SLE DA group for each sample was determined by the highest correlation score of the three correlations calculated. We then compared the average actual DA group scores (1.49, 2.01, 2.21 in Table 2) with each predicted GA group (S1, S2, and S3, respectively). We found that there were significant differences among these three average DA group scores (S1 vs S2, S1 vs S3, and S2 vs S3). Results suggested that metaSLEsig100 predicted DA groups correlated with the actual clinical SLEDAI group scores. Those groups with higher predicted SLE DA showed statistically significantly higher actual SLE DA group scores than those predicted to be lower SLE DA group (P ≤ 0.002). Thus, the SLEmetaSig100 centroid model can serve as a SLE biomarker that can improve SLE patient diagnosis and disease activity prediction.

**Table 2:** Association between SLE group prediction and actual SLE disease activities.

Predicated GSE65391 Group	S1	S2	S3
SLEDAI	0–2	3–7	>7
Group DA scores	1	2	3
Actual Average Group DA Score	1.49	2.01	2.21
SD	0.67	0.63	0.75
P values	≤0.002	≤0.002	≤0.002

## Discussion

SLE is a heterogeneous disease characterized by a wide spectrum of clinical manifestations and degrees of severity. A single gene-expression profiling study on SLE cannot capture the full molecular heterogeneity of SLE. Few studies on SLE gene expression signatures have progressed beyond the discovery phase because the retrospective validation of these signatures often focused on the association of DA at the group level within the same study [5, 8, 17].

We analyzed fifteen source datasets in this study from microarray platforms. In a typical transcriptional study of a disease model, a differential gene list is usually generated from a training set and is then validated in a test set. With both training and test sets from the same patient cohorts, a microarray study is often underpowered. Other common negative factors of microarray platform such as low data quality and high background noise interference can be introduced at different experimental and analysis stages.

In response to these challenges, we developed the EXALT method by combining related but independent studies into a meta-analysis to create a larger sample size and to produce a lower false discovery rate. EXALT is essentially a database containing thousands of gene expression signatures extracted from public gene expression database (e.g. GEO) and published studies that enable signature comparisons. We previously used this robust meta-analysis of gene expression profiles from hundreds of breast cancer datasets [11–13]. By using this approach, we discovered a novel and conserved gene expression signature that predicts metastasis risk in multiple cancers (breast, lung, and prostate cancer) [13]. Furthermore, we identified a new cancer metastasis-suppressor gene [25].

In this study, we applied our established high-throughput *in silico* method (EXALT) to identify and validate the meta-signature (SLEmetaSig100) based on fifteen available published studies. We demonstrated that heterogeneous signatures from thirteen training datasets containing 1,869 samples from heterogeneous whole blood PBMC or T cells could be systematically organized by their common data elements (i.e., intrinsic similarities and disease phenotypes) and assembled into a new signature data type called a meta-signature (S1 Table and Fig 1). We identified a meta-signature representing a common SLE gene expression profile consisting of 100 genes (SLEmetaSig100) that is capable of identifying SLE in 1,171 testing human samples from two independent datasets (Figs 3 and 4 and Table 2). These findings illustrate that there is a fundamental gene expression pattern conserved across immune cell types in SLE patients.

Besides SLEmetaSig100, several studies have shown that gene-expression signatures related to SLE DA [5, 8, 17]. We compared SLEmetaSig100 with a few representative signatures on SLE (Table 1). The main difference is that SLEmetaSig100 was identified based on much larger training sets from a unique meta-analysis (EXALT) and could stratify SLE at the individual patient level. The meta-signature represents heterogeneous cell populations that might result in weak overlap with any known SLE signatures (Table 1). Although the overlap in gene composition between SLEmetaSig100 and other published SLE signatures is small, they all contain a major functional component of the signature genes related to interferon (IFN) and/or IFN-induced genes that are included in the network of cytokine-cytokine receptor interaction. The gene functional groups and pathways of the meta-signature (SLEmetaSig100) are commonly known for their roles as seen in other SLE signatures or SLE studies (S2 Table).

The network of cytokine-cytokine receptor Interaction contains 18 SLEmetaSig100 genes (CCR7, CXCL10, CXCR4, CXCR5, CD40LG, TNFRSF1B, TNFRSF4, FLT3LG, IFNA1, IFNA17, IFNAR1, IFNB1, IL10, IL12A, IL12B, IL18, TNFSF13, TNFSF4) (S3 Fig, S2 Table). These cytokine genes often play direct effective roles in SLE pathogenesis through the regulation of systemic inflammation, local tissue damage, and immune modulation[26]. We confirmed a set of prevalent IFN-regulated transcripts in SLEmetaSig100 that are highly correlated with inflammation and IFN signaling pathways such as IFI16 IFIH1, IFNA1, IFNA17, IFNAR1, IFNB1, IFNG, IL10, IL12A, IL12B, IL18, IL‑1R1, and IL-21. The dysregulation of IFN family genes (IFNA1, IFNA17, IFNAR1, IFNB1) is dominantly pervasive, and their protein and gene expression profiles may serve as markers of disease activity and severity [26–29].

Besides the overexpressed IFN-inducible genes, SLEmetaSig100 also contains DNA sensor genes as described in the innate immunity DNA-sensing model (Fig 2) such as cyclic GMP-AMP Synthase (MB21D1 or cGAS), IFI16, and Toll-like receptors (TLR)s that are required for cell proliferation and for mounting an appropriate immune response to either a pathogen or cellular/tissue damage[22]. TLR3, TLR5 and TLR7/8/9 have been reported as facilitating SLE pathogenesis [30] (S1 Fig, S2 Table). These different TLRs provide distinct or synergistic contributions. For example, the expression levels of TLR2 and TLR4 mRNAs in SLE patients’ PBMCs are much higher than those in healthy subjects [31], and the expression of TLR3 mRNA increases with the progression of lupus nephritis [32, 33] while downregulation of TLR2 or TLR4 can decrease the production of autoantibodies and attenuates the development of renal injury in lpr mutation-induced murine lupus[34].

Other DNA sensor genes in the SLEmetaSig100 signature are key enzymes involved in breakdown of DNA including nucleases such as DNASE1, DNASE1lL3, TREX1, and TREX2. Importantly, a loss-of-function variant of DNASE1L3 causes a familial form of SLE. Mutations in TREX1 are associated with familial chilblain lupus and are also associated with the inflammatory disorder Aicardi-Goutieres syndrome. The SLEmetasig100 emphasizes the importance of including DNA processing pathways, which may capture the contributions of proteostasis and ER stress to SLE pathogenesis.

Lupus nephritis is a frequently seen complication in patients with SLE and is known to significantly reduce the survival of SLE patients. A hallmark of lupus nephritis is the renal inflammation caused by deposition of autoimmune complexes to kidney glomeruli. There are four SLEmetaSig100 genes (NFAIP3, IRAK4, MYD88, TLR4) in NF-kappa B signaling pathway (S2 Fig, S2 Table) that have been implicated in the pathogenesis of lupus nephritis[35] coupled with upregulation of inflammatory cytokines [36, 37].

Previous SLE signatures have been essentially equivalent in correlation to SLE DA except for the INFγ signature (Table 1). However, SLEmetaSig100 not only correlates with SLE DA but also provides a prospective prediction method that can improve SLE patient diagnosis, a capability that has not been demonstrated in any other SLE signature (Table 1, Table 2). Our result suggests that SLEmetaSig100 is capable of prospectively applying to individual patients with high PPV (97%-99%), specificity (85%-84%), and sensitivity (60–84%) (Fig 4). In the meantime, we also observed that SLEmetaSig100 has a low NPV rate (20%, Fig 4) which is the percentage of patients with a true negative test result who do not have the disease (SLE), suggesting that SLEmetaSig100 may not be suitable to predict healthy status.

Our approach may provide a new SLE biomarker for clinical diagnosis, classification and monitoring. Previous signatures have correlated with DA at the cohort level (retrospective stratification), such as IFN or plasmablast signatures (Table 1); however, they did not demonstrate the capability to predict SLE status and DA association in individual SLE patients.

A limitation of the current study is that we only tested SLEmetSig100 in samples of healthy and SLE subjects. Heterogeneous cell types with limited clinical attributes and follow-up information could hamper the training process and interpretation of our meta-analyses. There is no test data set available showing the correlation between the SLEmetaSig100 profile and a manifestation of SLE or another autoimmune disease. Treatment status was not accessible in the meta-data of the training and testing sets. Therefore, it may be possible that the SLE patients with signatures that were most similar to healthy controls were responding well to therapy. The capacity for SLEmetasig100 to predict early treatment response will be an important future application as well as its ability to distinguish SLE from other autoimmune disorders that may overlap in clinical presentation.

Future studies on SLEmetaSig100 with consecutive blood sampling from the same patient would allow us to better measure SLEmetSig100’s performance by tracking disease activity and response to treatment over time. More importantly, a more controlled training set would allow us to improve our meta-signature’s predictive ability to distinguish SLE profiles from those of other autoimmune disorders.

In summary, our finding supports the potential application of SLEmetSig100 as a promising biomarkers in clinical practice with an acceptable specificity and sensitivity. Biomarkers that can prospectively predict occurrence and frequency of flares will be of great clinical value in clinical practice [38]. The data mining nature of this study provides a foundation to further identify and validate more flare predictors. Additionally, the SLEmetaSig100 may also inform future study design to identify novel genes in SLE pathogenesis, classifiers, and early predictors of DA scores.

## Supporting information

S1 Fig Toll-like receptor signaling pathway. SLEmetaSig100 includes eight Toll-like receptors (TLR) genes (TLR1, TLR2, TLR3, TLR4, TLR5, TLR7, TLR8, and TLR9). Most of TLRs are up-regulated (TLR1, TLR2, TLR4, TLR5, TLR7, and TLR8) while two TLRs (TLR3 and TLR9) are down-regulated in SLE disease conditions.
(TIF)

S2 Fig NF-kappa B signaling pathway. NF-kappa B signaling pathway. There are four up-regulated SLEmetaSig100 genes (NFAIP3, NFIRAK4, MYD88, TLR4) and one down-regulated gene (NFKB1) in the NF-kappa B signaling pathway that are also present in the TLR signaling pathway as expected. As a negative regulator protein, NFKB1 is controlled by various mechanisms of post-translational modification and subcellular compartmentalization as well as by interactions with other cofactors or co-repressors.
(TIF)

S3 Fig Cytokine-cytokine receptor interaction. The network of cytokine-cytokine receptor Interaction contains 18 SLEmetaSig100 genes (CCR7, CXCL10, CXCR4, CXCR5, CD40LG, TNFRSF1B, TNFRSF4, FLT3LG, IFNA1, IFNA17, IFNAR1, IFNB1, IL10, IL12A, IL12B, IL18, TNFSF13, TNFSF4). Most cytokine genes are up-regulated (S1 Table) like IFN, IFN responsive genes, or chemokines except four down-regulated genes (CCR7, CXCR5, FLT3LG, and IL12A).
(TIF)

S1 Table Overview of SLE data sets on gene-expression profiles. (XLSX)

S2 Table Gene annotation for the SLE signature. (XLSX)
