# Supplementary figures and images for: Identification of a gene-expression predictor for diagnosis and personalized stratification of lupus patients
Source: PLoS One. 2018 Jul 5;13(7):e0198325. doi: 10.1371/journal.pone.0198325 (PMC6033382; doi:10.1371/journal.pone.0198325)

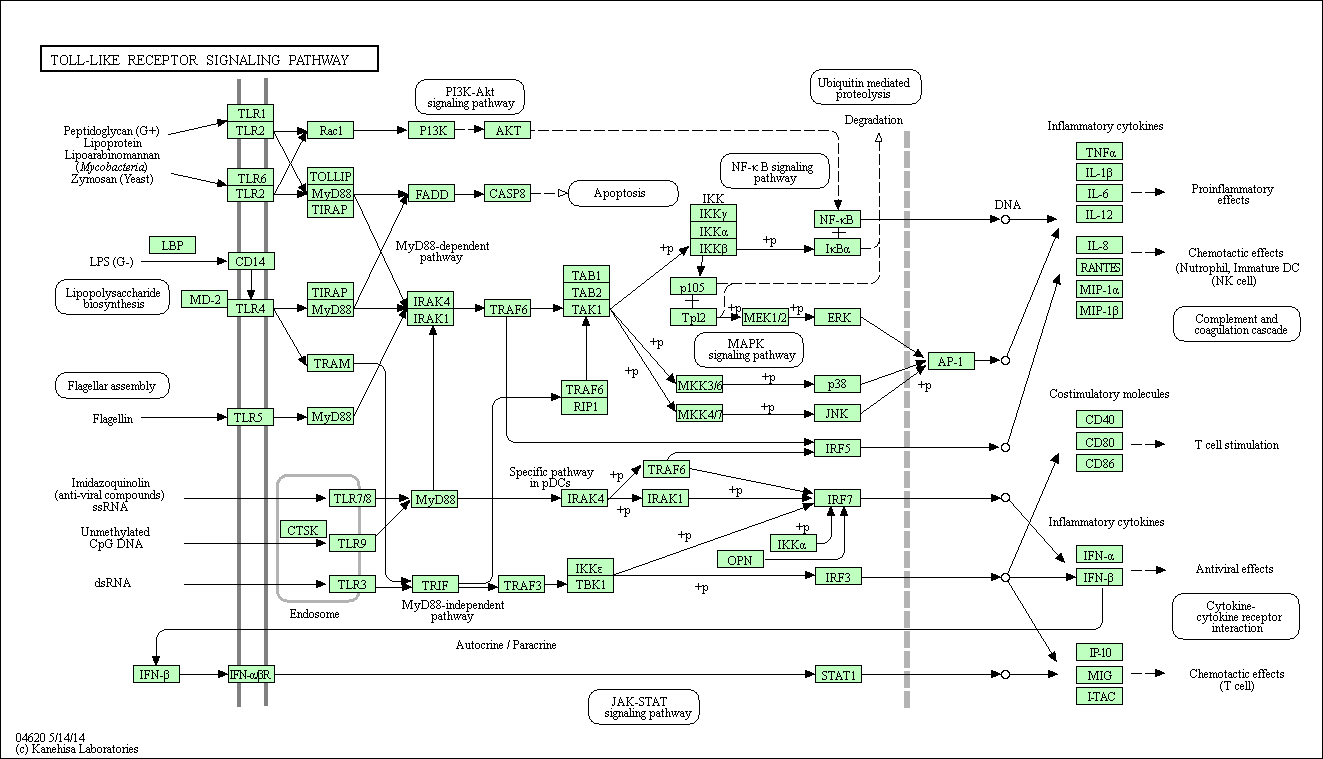

Supplement: S1 Fig — SLEmetaSig100 includes eight Toll-like receptors (TLR) genes (TLR1, TLR2, TLR3, TLR4, TLR5, TLR7, TLR8, and TLR9). Most of TLRs are up-regulated (TLR1, TLR2, TLR4, TLR5, TLR7, and TLR8) while two TLRs (TLR3 and TLR9) are down-regulated in SLE disease conditions. (TIF) [file pone.0198325.s001.tif]

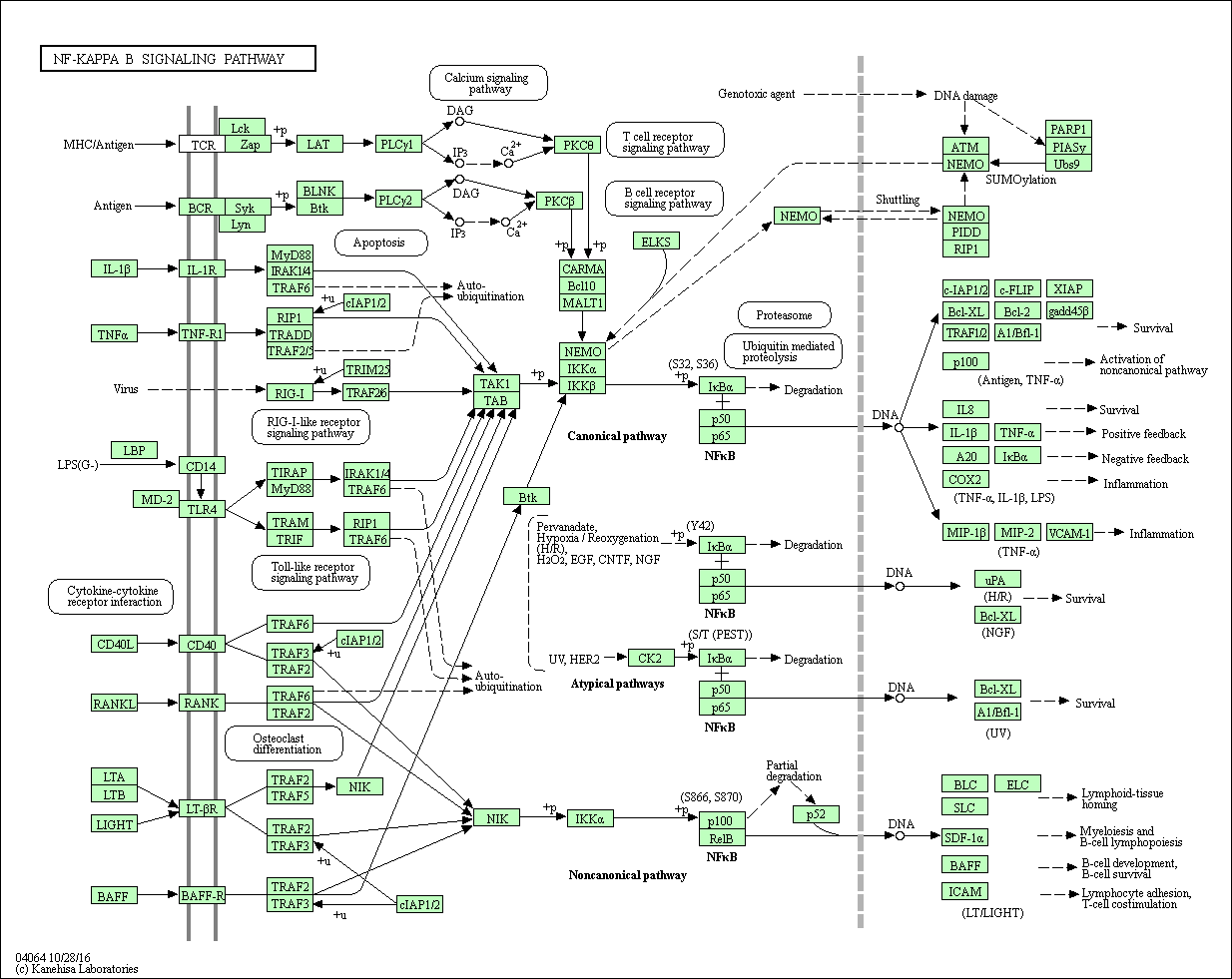

Supplement: S2 Fig — NF-kappa B signaling pathway. There are four up-regulated SLEmetaSig100 genes (NFAIP3, NFIRAK4, MYD88, TLR4) and one down-regulated gene (NFKB1) in the NF-kappa B signaling pathway that are also present in the TLR signaling pathway as expected. As a negative regulator protein, NFKB1 is controlled by various mechanisms of post-translational modification and subcellular compartmentalization as well as by interactions with other cofactors or co-repressors. (TIF) [file pone.0198325.s002.tif]

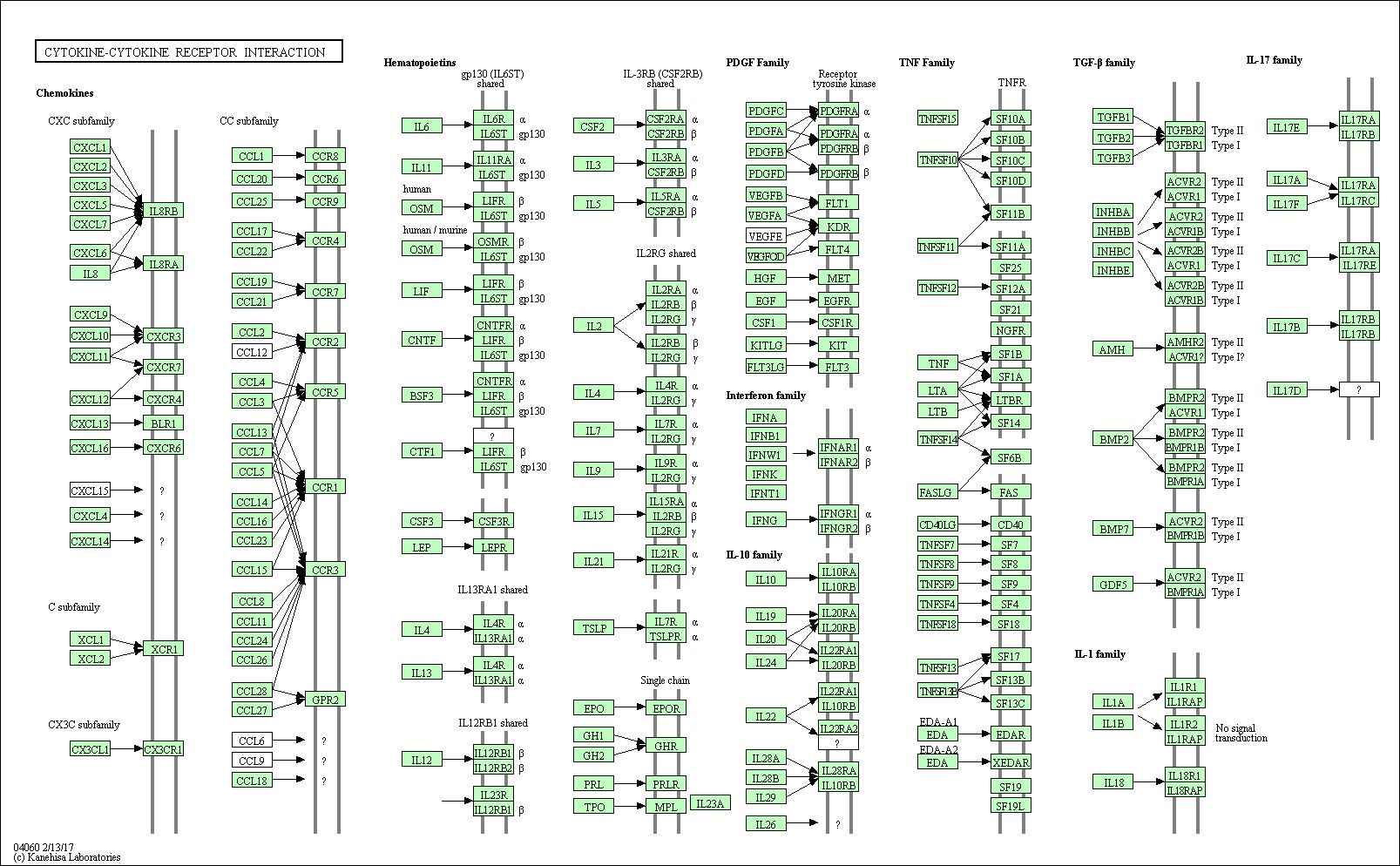

Supplement: S3 Fig — The network of cytokine-cytokine receptor Interaction contains 18 SLEmetaSig100 genes (CCR7, CXCL10, CXCR4, CXCR5, CD40LG, TNFRSF1B, TNFRSF4, FLT3LG, IFNA1, IFNA17, IFNAR1, IFNB1, IL10, IL12A, IL12B, IL18, TNFSF13, TNFSF4). Most cytokine genes are up-regulated (S1 Table) like IFN, IFN responsive genes, or chemokines except four down-regulated genes (CCR7, CXCR5, FLT3LG, and IL12A). (TIF) [file pone.0198325.s003.tif]
